# Supplementary material for: Drug Safety During Breastfeeding: A Comparative Analysis of FDA Adverse Event Reports and LactMed®
Source: Pharmaceuticals (Basel). 2024 Dec 9;17(12):1654. doi: 10.3390/ph17121654 (PMC11728675; doi:10.3390/ph17121654)
Supplement: Supplementary file 1 [file pharmaceuticals-17-01654-s001.zip › pharmaceuticals-3344082-supplementary.pdf]

pharmaceuticals

MDPI

Table S1. Overview of the available information on adverse infant events in the FAERS database and in LactMed® for the 25 selected products.

N: Nervous system	FAERS	LactMed®
Buprenorphine	Bradycardia<br>Coma scale abnormal<br>Drug withdrawal syndrome<br>Neonatal<br>Hypoglycemia<br>Hypotension<br>Irritability<br>Lethargy<br>Miosis<br>Poor feeding infant<br>Selective eating disorder<br>Somnolence<br>Sudden death	Agitation<br>Drowsiness<br>Drug withdrawal<br>Frequent yawning<br>Hyperactive Moro reflex<br>Insomnia<br>Lower milk intake<br>Lower weight gain<br>Myoclonic jerks<br>Opioid abstinence<br>Poor feeding<br>Pupillary dilation<br>Sneezing<br>Sweating<br>Tremors
Lamotrigine	Abdominal pain<br>Abnormal loss of weight<br>Apathy<br>Bradyarrhythmia<br>Cyanosis neonatal<br>Ecchymosis<br>Eczema<br>Failure to thrive<br>Fatigue<br>Feeding disorder<br>Fluid intake reduced<br>Hepatic enzyme increased<br>Hyperbilirubinemia neonatal<br>Hypotonia neonatal<br>Hypovolemic shock<br>Infantile apnea<br>Irritability<br>Jaundice<br>Laryngomalacia<br>Lethargy<br>Liver disorder<br>Malnutrition<br>Nausea<br>Neonatal hypoxia<br>Neutropenia<br>Normochromic normocytic<br>Anemia<br>Poor feeding infant<br>Poor weight gain neonatal<br>Rash<br>Rash maculo-papular	Anemia<br>Apneic episode<br>Drowsiness<br>Drug withdrawal<br>Elevated liver enzymes<br>Elevated platelet counts<br>Feeding problems<br>Gangrene<br>Gastrointestinal symptoms<br>Heart murmur<br>Hypotonia<br>Icterus prolongatus<br>Irritability<br>Jaundice<br>Liver damage<br>Loss of appetite<br>Neuromotor hyperexcitabil-<br>ity<br>Persistent crying<br>Rash<br>Retractive breathing<br>Sedation<br>Transient neutropenia<br>Weight loss

	Rhinorrhea<br>Selective eating disorder<br>Skin discoloration<br>Sleep disorder<br>Somnolence<br>Stridor<br>Supraventricular Extrasystoles<br>Thrombocytosis<br>Urticaria<br>Vomiting	
Levetiracetam	Anemia neonatal<br>Heart rate increased<br>Infantile apnea<br>Cyanosis neonatal<br>Blood bilirubin increased<br>Failure to thrive	Drowsiness<br>Hypotonia<br>Poor feeding infant<br>Poor weight gain<br>Sedation<br>Vomiting<br>Weight loss<br>Withdrawal seizures
Acetaminophen	Abdominal distension<br>Acute hepatic failure<br>Asthma<br>Blood pressure abnormal<br>Capillary nail refill test abnormal<br>Coagulopathy<br>Crying<br>Drug-induced liver injury<br>Erythema<br>Gastrointestinal hemorrhage<br>Hemoglobin decreased<br>Heart rate increased<br>Hepatomegaly<br>Hypoglycemia<br>Irritability<br>Jaundice<br>Livedo reticularis<br>Metabolic acidosis<br>Poor feeding infant<br>Pulse abnormal<br>Pyrexia<br>Respiratory distress<br>Respiratory syncytial virus bronchiolitis<br>Selective eating disorder<br>Shock<br>Skin exfoliation<br>Staphylococcal infection	Asthma<br>Maculopapular rash on the upper trunk and face<br>Wheezing

	Upper gastrointestinal hemorrhage<br>Vomiting<br>Wheezing	
Nicotine	Dyspnea	Reduction in the heart rate<br>Sudden infant death syndrome
L: Antineoplastic and immunomodulating agents		
Certolizumab pegol	Agitation<br>Fungal infection<br>Hematochezia<br>Hematoma<br>Irritability<br>Nervousness<br>Rash<br>Restlessness<br>Selective eating disorder<br>Tongue disorder<br>Wound	Candida infection<br>Upper respiratory infection<br>Vomiting
Adalimumab	Nasopharyngitis<br>Irritability<br>Intestinal hemorrhage<br>Gastrointestinal disorder	None reported
Etanercept	Blood bilirubin abnormal<br>Blood glucose decreased<br>Death<br>Dermatitis atopic<br>Diarrhea<br>Disturbance in attention<br>Dyslexia<br>Enterocolitis<br>Feeding intolerance<br>Gastroesophageal reflux Disease<br>Gross motor delay<br>Hemoglobin decreased<br>Jaundice neonatal<br>Lung disorder<br>Nasopharyngitis<br>Pneumonia<br>Rash macular<br>Respiratory tract congestion<br>Seborrheic dermatitis<br>Selective eating disorder<br>Viral infection	High-pitched crying<br>Rash

Infliximab	Weight decreased<br>Weight gain poor<br>White blood cell count increased<br><br>Hematochezia<br>Jaundice<br>Lactose intolerance<br>Lower respiratory tract infection<br>Lymph gland infection<br>Malaise<br>Poor feeding infant<br>Selective eating disorder	None reported
Tacrolimus	Diarrhea<br>Intraventricular hemorrhage neonatal<br>Neonatal asphyxia<br>Neonatal respiratory distress syndrome<br>Pneumothorax	None reported
A: Alimentary tract and metabolism		
Insulin	Gastroesophageal reflux disease<br>Hematochezia<br>Necrotizing colitis	None reported
Omeprazole	Diarrhea<br>Faeces discolored<br>Zinc deficiency	None reported
Ondansetron hydrochloride	Crying<br>Insomnia<br>Irritability<br>Sluggishness	None reported
Mesalamine	Abdominal pain upper<br>Blood albumin abnormal<br>Candida infection<br>Colitis<br>Diarrhea<br>Hematochezia<br>Hemoglobin abnormal<br>Pyrexia<br>White blood cell count increased	Diarrhea<br>Thrombocytosis<br>Thrombosis
Metformin hydrochloride	Tremor	None reported

J: Antiinfectives for systemic use		
Zanamivir	Abnormal faeces<br>Decreased appetite	No information
Amoxicillin/Clavulanic acid	Agitation<br>Clostridium difficile colitis<br>Conversion disorder<br>Diarrhea<br>Enterocolitis<br>Eye swelling<br>Gastrointestinal pain<br>Hematochezia<br>Oral candidiasis<br>Pyrexia<br>Rash maculo-papular<br>Rectal hemorrhage<br>Vomiting	Constipation<br>Diarrhea<br>Elevated liver enzymes (AST and ALT)<br>Generalized urticaria<br>Rash<br>Restlessness
Tenofovir disoproxil fumarate	Thrombocytopenia	Diarrhea
Lamivudine	Sudden infant death syndrome	Sudden infant death syndrome
Emtricitabine/Tenofovir	-	Diarrhea
R: Respiratory system		
Omalizumab	Anemia<br>Anaphylactic reaction<br>Cough<br>Decreased appetite<br>Eczema<br>Eye oedema<br>Gastroesophageal reflux disease<br>Nasopharyngitis<br>Oral fungal infection<br>Otitis externa<br>Poor quality sleep<br>Pyrexia<br>Rash erythematous<br>Seborrheic dermatitis<br>Swelling face	None reported
Fluticasone propionate/Salmeterol xinafoate	Middle insomnia<br>Poor feeding infant	None reported
Cetirizine hydrochloride	Abnormal faeces<br>Cyanosis	Bruising<br>Colicky symptoms

	Floppy infant<br>Lethargy<br>Oxygen saturation decreased<br>Rash<br>Respiratory arrest<br>Somnolence	Constipation<br>Drowsiness<br>Fever<br>Irritability<br>Poor feeding<br>Rash<br>Refusing of the breast<br>Sedation
Elexacaftor\Ivacaftor\Tezacaftor	Alanine aminotransferase increased<br>Aspartate aminotransferase increased<br>Bronchiolitis<br>Hyperinsulinemia<br>Hypoglycemia neonatal<br>Jaundice neonatal<br>Neonatal respiratory distress<br>Pancreatic failure<br>Sepsis neonatal<br>Sweat test abnormal<br>Transient tachypnoea of the newborn<br>Viral infection	Bilirubin abnormalities<br>Liver enzyme abnormalities<br>Low sweat chloride
Budesonide	Adrenocortical insufficiency neonatal<br>Dermatitis<br>Eczema<br>Neutropenia	None reported
